# Space-Filling Curve Resistor on Ultra-Thin Polyetherimide Foil for Strain Impervious Temperature Sensing

**DOI:** 10.3390/s21196479

**Published:** 2021-09-28

**Authors:** Korbinian Rager, David Jaworski, Chresten von der Heide, Alexander Kyriazis, Michael Sinapius, Iordania Constantinou, Andreas Dietzel

**Affiliations:** 1 Institut für Mikrotechnik, Technische Universität Braunschweig, 38124 Braunschweig, Germany; d.jaworski@tu-braunschweig.de (D.J.); c.von-der-heide@tu-braunschweig.de (C.v.d.H.); i.constantinou@tu-braunschweig.de (I.C.); a.dietzel@tu-braunschweig.de (A.D.); 2 Institut für Mechanik und Adaptronik, Technische Universität Braunschweig, 38106 Braunschweig, Germany; a.kyriazis@tu-braunschweig.de (A.K.); m.sinapius@tu-braunschweig.de (M.S.)

**Keywords:** ultra-thin sensor foil, space-filling curve, strain impervious temperature sensor, composite material monitoring, sensor integration

## Abstract

Monitoring process parameters in the manufacture of composite structures is key to ensuring product quality and safety. Ideally, this can be done by sensors that are embedded during production and can remain as devices to monitor structural health. Extremely thin foil-based sensors weaken the finished workpiece very little. Under ideal conditions, the foil substrate bonds with the resin in the autoclaving process, as is the case when polyetherimide is used. Here, we present a temperature sensor as part of an 8 µm thick multi-sensor node foil for monitoring processing conditions during the production and structural health during the lifetime of a construction. A metallic thin film conductor was shaped in the form of a space-filling curve to suppress the influences of resistance changes due to strain, which could otherwise interfere with the measurement of the temperature. FEM simulations as well as experiments confirm that this type of sensor is completely insensitive to the direction of strain and sufficiently insensitive to the amount of strain, so that mechanical strains that can occur in the composite curing process practically do not interfere with the temperature measurement. The temperature sensor is combined with a capacitive sensor for curing monitoring based on impedance measurement and a half-bridge strain gauge sensor element. All three types are made of the same materials and are manufactured together in one process flow. This is the key to cost-effective distributed sensor arrays that can be embedded during production and remain in the workpiece, thus ensuring not only the quality of the initial product but also the operational reliability during the service life of light-weight composite constructions.

## 1. Introduction

Nowadays, products containing electronic components are taking an ever-larger share of the market for consumer and industrial articles, in particular in the field of small and portable devices combining an increasing number of functions [1]. In the manufacturing industry, in health care and in the consumer sectors, sensing capabilities that monitor processes, body functions, environmental conditions or the structural health of constructions are of growing importance. Sensors designed in the form of flexible or even stretchable foils appear to be advantageous in many fields of application [2]. Foil-based strain sensor arrays for medical and technical applications based on thin film metallic conductors have been researched in great depth in our group [3,4,5]. Recently, multifunctional foil-based inlays for crack-sensing in composite structures have been developed [6].

An important parameter in almost all areas of measurement and sensing is temperature, because it is an almost unavoidable influencing factor, and sensors for other physical parameters often have to be compensated for temperature shifts. To allow compensation, temperature sensors can be integrated into constructions that are fully flexible or only yield slightly under load, also taking into account the variable strain that may interfere with the temperature sensors.

Widely used measuring methods for temperature are thermocouples and resistance thermometers. A thermocouple consists of two different metals that are connected at the point to be measured. Due to the Seeberg effect, a voltage can be measured that depends on the temperature difference between the measuring point and the reference point. It is beneficial that the characteristic curves of such sensors are approximately linear, but it is detrimental that these sensors have a low accuracy and low signal-to-noise ratio compared to other measuring principles. The resistance thermometer can be built of only one type of material that changes in electrical resistivity with temperature as described by the temperature coefficient. Resistance thermometers come in the form of PTC and NTC (positive and negative temperature coefficient) thermistors. While the PTC thermistors’ resistance rises, the NTC thermistors’ resistance drops on increasing temperatures [7]. Resistance thermometers are simpler in structure and thus allow more variety in the construction. Therefore, this concept is very well suited to be realized as a thin film sensor [8]. A flexible anemometer based on thin film resistors fabricated on PI foil material for integration into an airplane wing has already been developed in our group [9]. PVDF, which is a non-typical substrate for microfabrication, was used as a carrier film for an integrated flexible sensor system [6]. Moreover, the use of polyetherimide (PEI) as an ideal substrate for foil sensors to be integrated in composite structures was demonstrated recently [10]. For monitoring processing conditions during the production or structural health monitoring during the lifetime of a construction, thin film sensors were integrated in carbon fiber-reinforced polymers (CFRP). Unlike most other applications, the change in resistance due to temperature is no longer orders of magnitude higher than the change in resistance due to strains that typically occur in these materials. Particularly, if the aim is to monitor the curing state using film-integrated impedance sensors, temperature effects and strain effects must be detected and compensated for as independently of each other as possible. While a bridge circuit in strain sensor technology can compensate for temperature effects, the compensation of strain effects in temperature sensor technology is more difficult, not least because strain is a variable that is generally described by a tensor. However, for curing monitoring, the measured variable temperature is of particular interest, since the change in permittivity often measured with film sensors exhibits a strong temperature dependence. Therefore, the addition of a reliable temperature measurement represents an interesting added value.

Several approaches to the problem of strain compensation in resistance thermometers can be found in the literature. These range from a sensor on stretchable material including stretchable cables [11] to a complex array consisting of several resistive sensors that can determine temperature and strain through intelligent interconnection [12]. There are also approaches to manufacture sensors directly on already curved surfaces by photolithography [13]. However, these methods are complex and usually require several materials and many manufacturing steps to achieve the desired effect. This paper describes a new approach of a resistance thermometer, in which an inherent compensation of strain is realized. This thermometer is fabricated in combination with a thin film strain sensor and a thin film coplanar impedance sensor to establish a sensor node for monitoring the curing of fiber-reinforced polymers. Particular attention was paid to ensuring that the sensor node is designed and manufactured as simply as possible.

## 2. Materials and Methods

### 2.1. Micro Fabrication

To generate a thin flexible substrate, a liquid precursor with a 10 wt % content of polyetherimide (PEI) dissolved in trichloroethanol is spin-coated on a 4″ glass wafer, which has been previously provided with a few nanometers thick adhesive ring of chromium. After evaporation of the solvent at 175 °C for 60 min, the remaining PEI solid film has a thickness of only 8 µm. As a next step, the sensor thin film, which consists of a chromium adhesion layer of 10 nm and a gold layer of 300 nm, is deposited by sputter deposition (Laborsystem LS 440 S, Ardenne Anlagentechnik GmbH, Dresden, Germany). The geometry of the sensor track is defined by a lithographic process, wherefore the ma-P1215 photoresist (micro resist technology, Berlin, Germany) is deposited on top of the gold layer via spin coating. Then, the resist is exposed to UV light through a mask, which specifies the desired structure of the conductive tracks and sensors. Afterwards, the areas that were exposed are removed with a developer solution (ma-D 331, micro resist technology, Berlin, Germany), leaving the photoresist in the form of the requested structures on the gold layer.

Following the lithographic process, the metallic coating is structured using a wet chemical process to define conductor paths. First, the gold layer is removed in a gold etching bath consisting of iodine–potassium iodide solution. In the second step, the chromium is also removed in an alkaline chromium-etching bath. Finally, the remaining photoresist is stripped with ethanol in a spin-rinse process to uncover the structures. In order to minimize the resistance of the sensor feed lines, they are coated with a 6 µm thick copper layer in an electroplating bath. Then, this layer is tin-plated in a Sur-Tin [14] bath to prevent oxidation and improve the solderability of the contact pads.

The sensors are cut out on the wafer using a contour scan with a femtosecond microstructuring workstation (microSTRUCT C, 3D-Micromac, Dresden, Germany) using a green pulsed laser beam (generated by Light Conversion PHAROS 15W (Vilnius, Lithuania), wavelength 515 nm at second harmonic, pulse duration 230 fs, repetition rate 100 kHz, scan speed 500 mm/sec, pulse energy 50 µJ, focusing lens Qioptiq Linos F-Theta Ronar lens 100 mm (Göttingen, Germany)). As a last step, 4 cables per sensor are soldered to the corresponding contact pads. This is done while the film is still on the rigid wafer. Then, a weakly self-adhesive film (Tecni-Tape, DISCO Corporation, Tokyo, Japan) is used to detach the sensors from the wafer, so they can be applied to the desired test object without damaging the fragile sensor structures.

### 2.2. Design of the Temperature Sensor as Part of a Multiple Sensor Node

The sensors for strain, temperature, and impedance measurement are manufactured together so that they are all made of gold/chromium thin-film structures. The temperature sensor shall create a sufficient electrical resistance change in dependence of temperature within a planar conductor track, while resistance change due to unavoidable strains shall be kept to a minimum. With a long conductor path with a resistance of 100 Ω or more, temperatures can be recorded with simple measuring instruments. The arrangement of this conductor path follows a space-filling curve (SFC), which is a variation of a Hilbert curve [15] known as a one-dimensional path that can completely cover a two-dimensional area without touching or overlapping. The Hilbert curve consists of the same number of straight equal-length segments oriented in two orthogonal directions. In our design, the space-filling curve is rounded, and there are no straight segments. This is to ensure that when the component to be measured is stretched or compressed, the direction of strain does not matter [16]. In addition, longitudinal and transverse contributions for each finite segment of the curve as given in (1) should at least partially cancel each other out.

(1) $$\frac{\Delta R}{R}={\left(\frac{\Delta R}{R}\right)}_{long.}+{\left(\frac{\Delta R}{R}\right)}_{trans.}$$

A strong reduction of strain influence can be assumed. Since the non-linear conductor tracks strongly interact mechanically with the PEI substrate, which causes rather complex electromechanical interactions, the sensor concept needs to be evaluated in experiments and simulations.

**Figure 1:**
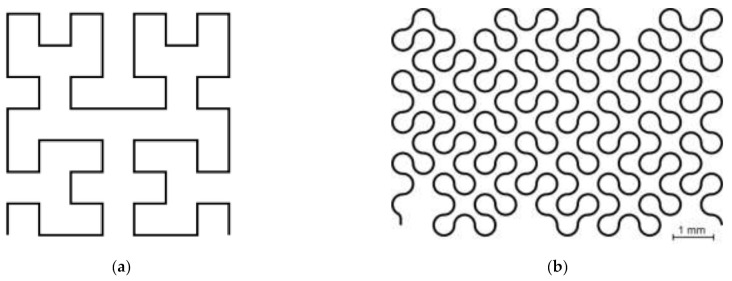
(**a**) Hilbert curve of 2nd order; (**b**) SFC as used in the temperature sensor.

A gold layer thickness of 300 nm and a track width of 50 µm result in a cross-sectional area of $1.5\times 10^{-5}\;{\mathrm{mm}}^{2}$. The target resistance is chosen to be 300 Ω, and with the specific resistance of $2.214\times 10^{-2}\;{\Omega \mathrm{mm}}^{2}/m$ (Gold), the length of the temperature measuring resistor is set to 203 mm, which must be distributed over as small an area as possible to ensure that the temperature is measured at a specific point. The SFC as shown in Figure 1 in comparison to the Hilbert curve consists of connected 90° circular segments with a radius of 250 µm. In this way, the conductor path of 203 mm length is contained in an area of about 5.5 × 8 mm.

**Figure 2:**
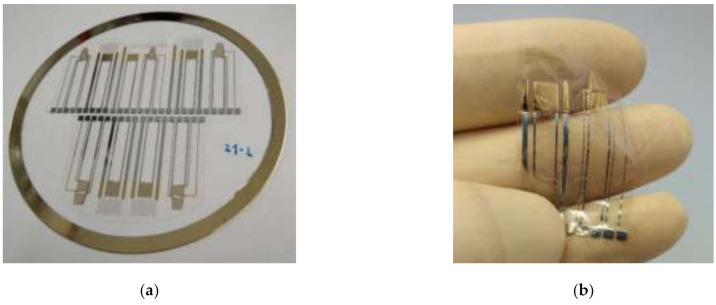

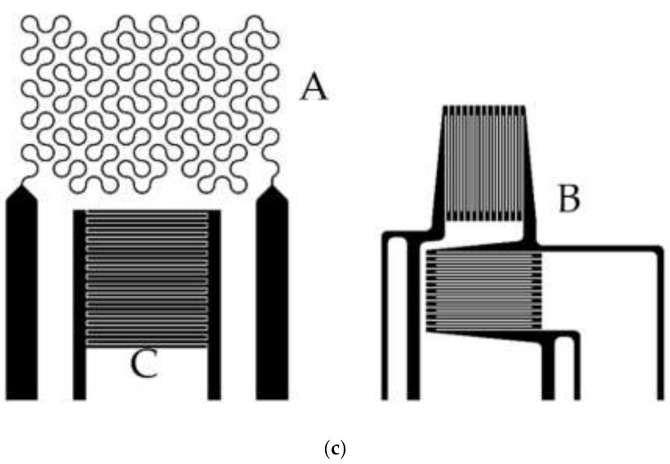
(**a**) Manufactured sensor nodes on a 4″ glass wafer, next to the sensor structures also the metallic coating ring for better adhesion of the PEI film is visible; (**b**) sensor node on PEI foil released from the carrier; (**c**) schematic of sensor node design, consisting of SFC-temperature sensor (A), two strain sensors in half-bridge circuit (B), and an interdigitated electrode capacitance (C).

The temperature sensor is part of a cure process and structural health monitoring sensor node. This also includes two orthogonal strain gauge sensors connected in a half bridge in order to eliminate the temperature influence and a coplanar electrode capacitance made of interdigitated structures for monitoring impedance as a measure for the cure state of the resin in a composite material. Figure 2 shows fabricated sensor nodes still attached to the 4″ glass wafer together with the schematic layout.

### 2.3. Simulation Methodology

In order to validate the isotropic strain sensitivity of the proposed sensor geometry, a thin three-dimensional numerical model in the finite element software COMSOL Multiphysics (version 4.3a) was established. The modules Solid Mechanics and AC/DC were used. Firstly, a mechanical model was used to obtain deformed sensor shapes for different load directions. Secondly, the deformed sensor shapes were used as an input in an electric model to analyze deformation-induced changes of the electrical resistance. The adhesion-enhancing chromium layer was neglected for reasons of computational effort. Discretization of the geometry was conducted by applying a mesh of prism elements. For the boundary conditions, we assumed that the deformation of the PEI layer fully transfers to the golden conductor. A biaxial displacement field that represents the stretched PEI foil underneath the golden part was applied to the lower surface of the geometry. The displacement field is defined by two linear functions according to (2) and (3):

(2) $$u_{1}\left(x\right)=\varepsilon_{i}\cdot x$$

(3) $$u_{2}\left(y\right)=-\nu_{PEI}\cdot \varepsilon_{i}\cdot y$$

$u_{1}$ and $u_{2}$ are the deformations, x and y are the coordinates of the reference configuration, $\nu_{PEI}$ is the Poisson ratio of PEI, and $\varepsilon_{i}$ is the applied strain. Three PEI strain cases were applied (0.5%, 1%, and 2% strain). In order to analyze the isotropy, different orientations with respect to the strain field were investigated (φ=0°…90°). In the electrical model, a current of 1 A was applied to the deformed geometry. Thus, the calculated voltage drop has the same numerical value as the resulting resistance of the cell. By referring the resistance of the loaded to the unloaded case, we obtain the relative change of resistance due to the strain. As a reference, the above described model was also applied to a simple straight conductor with the same cross-section and a length of 2 mm. The applied material properties are: Poisson ratio of PEI $\nu_{PEI}=0.44$, Poisson ratio of gold $\nu_{Au}=0.44$, Young’s modulus of PEI $E_{PEI}=2.9\;\mathrm{GPa}$, Young’s modulus of gold $E_{Au}=70\;\mathrm{GPa}$, electrical conductivity of gold 45.6 MS/m.

### 2.4. Setups and Procedures for the Experiments

The SFC sensors were calibrated while being placed between two glass wafers, forcing them into a flat shape. A thermocouple (307 Thermometer, K-Type thermocouple, CIE, New Taipei City, Taiwan) was placed on the glass wafer directly above the sensor with a heat-conducting paste and fixed with a heat-resistant adhesive tape. The thermocouple was calibrated before to 0 °C in iced water. The wafer sandwich was placed on top of a hotplate (PZ28-2, Harry Gestigkeit GmbH, Düsseldorf, Germany), which was heated up to 120 °C and then turned off. During cool down, resistance was repeatedly measured using a multimeter (UT131A, UNI-T, Opava, Czech Republic). In a next step, different degrees of strain were applied at room temperature by bending over metal tubes with different radii. The sensors were fixed to the tubes by means of a heat-resistant kapton foil cover to ensure that they take on the shape of the tube. The temperature of the metal tube was measured directly next to the sensor with the thermocouple after a settling time of 5 min. Different sensor orientations were realized by placement in orientations 0°, 45°, and 90° relative to the tube axis. Finally, temperature measurements were combined with a bending load. An oven (T 5042 BK, Heraeus, Hanau, Germany) was used for the experiments in which the bending setups were heated completely, and sensor signals were logged at temperatures between 120 °C and room temperature. The thermocouple was attached to the tube next to the sensors, which were tied around the tube and held in place by the cover foil. Thin cables were soldered to the sensor and led out through a gap in the oven door. The 4-wire measurement allows the resistance to be measured directly in the sensor without having to consider the resistance of the supply cables. The resistance was read out with a digital multimeter (Agilent, 34401A Digital Multimeter, Santa Clara, CA, USA). While heating up the oven, the temperature distribution inside cannot be assumed to be homogeneous. Therefore, the oven was heated up to 130 °C and then turned off. By cooling down slowly with the door closed, the temperature inhomogeneities in the measurement setup placed in the center of the oven are minimized. All sensors to be measured are arranged with equal distances to the thermocouple. Starting at 120 °C, the resistance was recorded every 5 Ω, until room temperature had been reached. In this way, a direct comparison can be made to the linear decrease in resistance measured without bending stress. In parallel, the resistance of strain gauge structures of the sensor nodes were read out for comparison.

## 3. Results and Discussion

### 3.1. Fabrication

As in a previous study [10], polyetherimide was chosen as the substrate material for the sensor. This material is particularly suitable for embedding in composite structures with epoxy resin, as it does not significantly weaken the structure of fiber composite components. It is worth mentioning that with the use of trichloroethanol as a solvent for PEI, it becomes possible to produce thin polymer foils by spin coating. Trichloroethanol is characterized by a significantly lower health hazard compared to other possible solvents and was previously used to produce a precursor for polyamide [17].

### 3.2. Simulation of Strain-Induced Resistance Change

**Figure 3:**
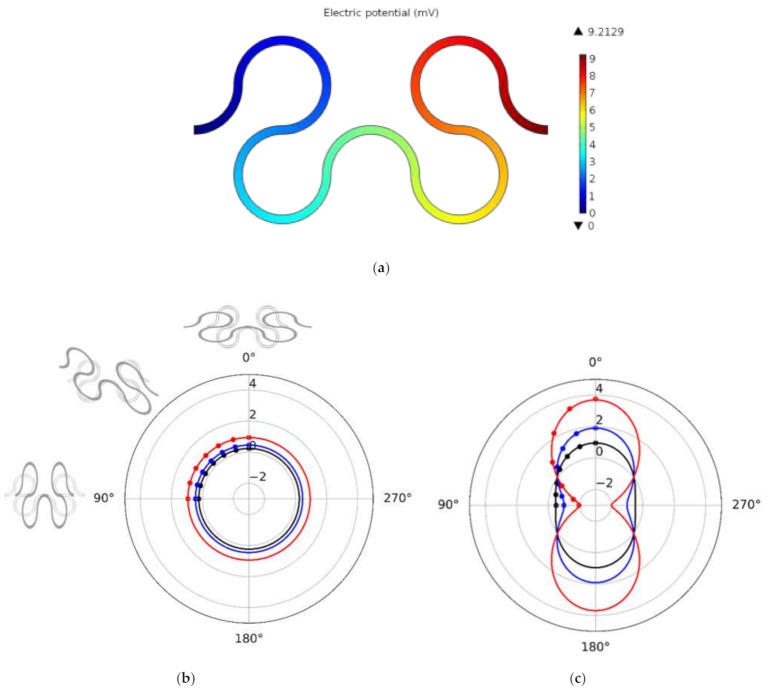
(**a**) Electric potential in the SFC elementary cell sensor as obtained by FEM simulation for a 90° load case and 1% of strain; (**b**) ΔR/R in dependence of the direction of applied strain in a polar coordinate view for the SFC and (**c**) for a linear gauge with same conductor cross-section for comparison. Dots represents data obtained by FEM simulation and lines are obtained by symmetry considerations and interpolation. The color indicates the magnitude of strain (black = 0.5%; blue = 1%; red = 2%). The images at 0°, 45°, and 90° illustrate the deformation pattern for these load directions.

In order to avoid unnecessary computing efforts, only an elementary cell, which basically always repeats itself being connected to the SFC sensor, was considered in the FEM modeling. This elementary cell has the same proportion of quarter circle conductor segments oriented in two orthogonal directions in the plane and therefore should react with the same sensitivity regardless of the direction of the applied strain. The cell includes a conductive track with a thickness of 300 nm and a width of 50 µm, which is fixed on an 8 µm thick PEI film that is stretched in the directions from 0° to 90°. The same PEI film with a straight conductor, also with a thickness of 300 nm and a track width of 50 µm, serves as a reference similar to the strain gauge structures that were used in the experiments described below. Figure 3a shows an example for the voltage drop along an SFC elementary cell for the stretching direction of 90°. In addition, in Figure 3, the change of resistance ΔR/R vs. the stretching directions is depicted in polar coordinates (b) for the SFC cell and (c) for the straight gauge cell. For reasons of symmetry, only angles from 0° to 90° needed to be calculated in the simulation. As it has been expected for the SFC cell, the simulation results show that the resistance change is invariant toward the stretching direction—in contrast to the straight conductor, which has a highly orthotropic behavior. The change of resistance of the straight conductor is characterized by a cosine function. It is notable that the average relative resistance change of the straight cell is equal to the direction-independent value of the SFC.

The simulations show that for the SFC sensor, the resistance (in any direction) increases by 0.65% at 1% of the applied strain compared to the unloaded sensor, whereas the resistance of the strain gauge at 0° increases by 1.81% at 1% of applied strain compared to the unloaded sensor. This is leading to a factor of 2.8 in advance for the SFC sensor in 0° strain direction.

### 3.3. Calibration of the SFC Sensor

**Figure 4:**
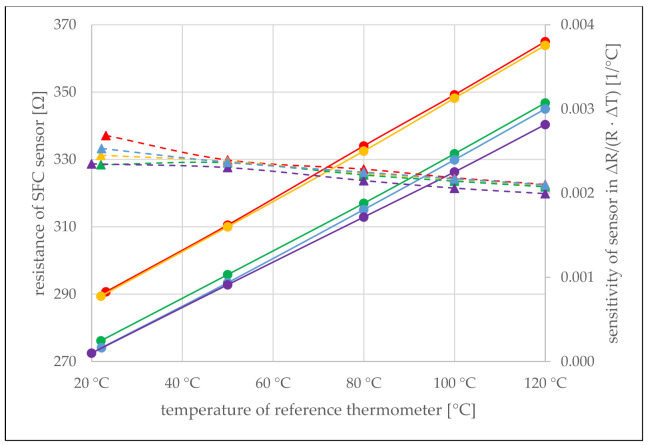
Linear temperature response of five different SFC sensors (the measuring points are indicated by circles) after release from the glass substrate and heating to 130 °C (burn-in). Practically identical sensitivities of about 0.002 1/°C are obtained (the values calculated from the measuring points are indicated by triangles connected by dashed lines).

Sensor signals were recorded right after micro fabrication while still attached to the glass wafer substrate. The measurement was repeated after the sensors were detached from the glass wafer, inserted into the test device, and connected. For both measurements, the temperature was measured in parallel with a calibrated thermocouple after the temperature has settled for several minutes. It is noticeable that the resistance values were 0.8% to 1.6% lower after sensors were detached from the carrier substrate. This is most likely due to residual stresses that develop during the micro fabrication, which can relax when the sensor is removed from the wafer. To prevent further stress relaxation effects, each individual sensor was preheated at a high temperature, close to a maximum of 139 °C, which is set by the melting point of the solder connections of the attached cables but still well below the glass transition temperature of PEI at 217 °C. Then, the heating was switched off, and the assembly cooled down slowly and steadily. At 120 °C, 100 °C, 80 °C, 50 °C, and room temperature, the resistance values of the sensor were recorded. Upon reaching room temperature, the resistance value was 2–3% lower than immediately after release from the wafer. This behavior can be explained by the last residues of solvents in the PEI film that were released and by the relaxation of residual stresses in the film, which will be discussed in more detail in the section on temperature measurement under strain influence. Nevertheless, a good linear response to temperature with practically identical slopes was observed for all five sensors. As can be seen in Figure 4, the sensors only differ by an offset value, which results from the manufacturing process of the sensors. However, the temperature sensitivity calculated for all measurement points based on the resistance value at 20.0 °C room temperature results in an almost constant value (nearly horizontal lines) of about 0.002 °C^−1^.

### 3.4. Bending Sensitivity

**Figure 5:**
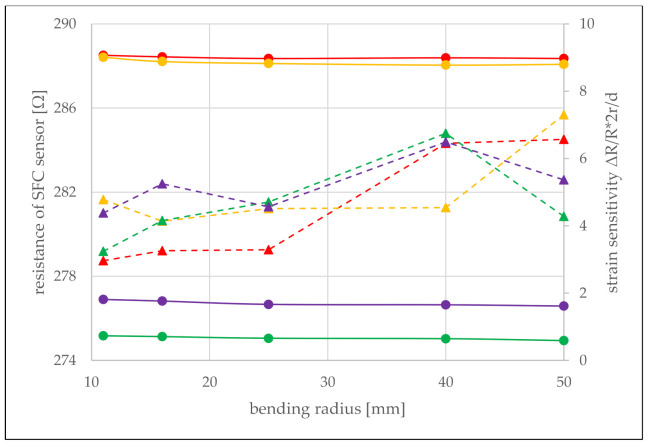
Resistance as a function of bending stress measured at 20.0 °C (circles). Similar strain sensitivities around 5 (the values calculated from the measuring points are indicated by triangles connected by dashed lines) are obtained. The strong fluctuation of the strain sensitivity is due to the measurement inaccuracies with very small measured values.

The recorded room temperature slightly varied in our laboratory during the experiments (±2 °C). Therefore, the measured resistance values for each bending radius were corrected for exactly 20.0 °C using the previously obtained calibrations, so that measurements at different radii can be directly compared. Figure 5 shows that the resistances almost do not vary with the bending loads. Only in the case of very strong bends with a radius r≤ 15 mm, a slight increase in resistance of ΔR=0.3 Ω is noted. This strain-induced change in resistance compares to the resistance change resulting from a temperature change of less than half a degree, which is below the desired resolution of the temperature measurement. The sensors were also measured in three different orientations: 0°, 45°, and 90° rotated against the bending axis with practically identical results. This confirms that the SFC sensor does not respond to the direction of strain loading as predicted by the simulations and shown in Figure 3b. Assuming that the PEI film behaves as described in the classical beam bending theory, the neutral line, which is neither compressed nor stretched, is located in the center of the foil cross-section. Therefore, the distance of the sensor from the neutral line is half the thickness d=8 µm of the PEI foil, and the strain can be calculated as ε=d/2r, and the strain sensitivities are given as ΔR/R·2r/d. As can be seen from Figure 5, a low strain sensitivity of about 5 was obtained for all four sensors (one sensor broke during this experiment).

### 3.5. Temperature Measurement under the Influence of Strain

**Figure 6:**
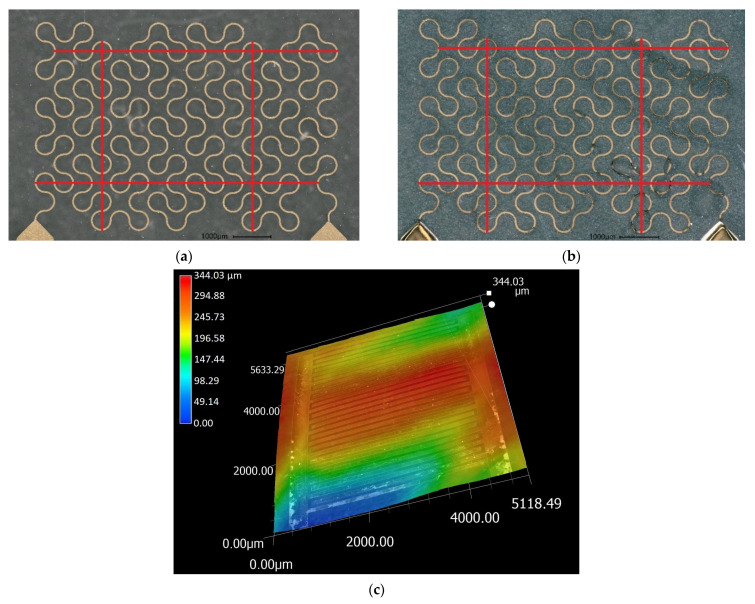
(**a**) Sensor node still attached to the wafer; (**b**) sensor node after detachment and extended burn-in, length measurements between the indicated lines reveal a shrinkage of 1% in all directions; (**c**) waviness of the sensor node foil as obtained by digital microscopy (VHX5000, Keyence) in the area of the capacitive sensor fixed with adhesive tape to a flat waver surface.

Initial combined strain and temperature measurements showed that during heating cycles, regardless of the applied load, the electrical resistance of each sensor continued to decrease with each cycle. This could be eliminated by applying 20 heating cycles starting at room temperature and reaching a maximum temperature, which was increased with each next cycle. In the final cycle, the sensor was heated up to 175 °C. In all further cycles not exceeding 130 °C, no further change in the resistance was observed. However, a microscopic examination of sensors after this extended burn-in procedure revealed that the film surface had taken on a wavy shape as a result of strain relaxation. Figure 6 shows this waviness, the shrinkage of the film, and also the accompanying lateral shrinkage.

**Figure 7:**
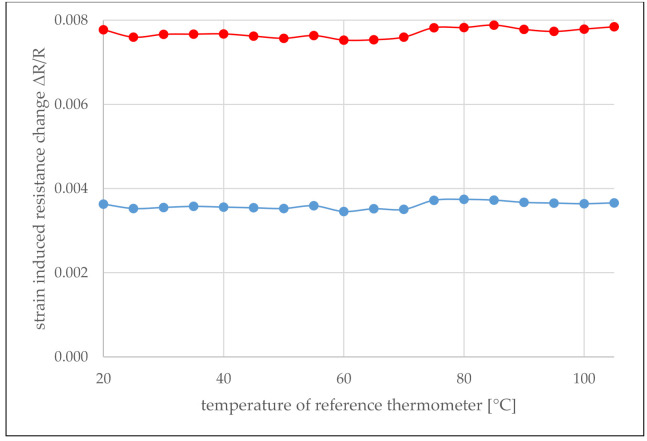
Strain-induced ΔR/R in dependence of temperature for the SFC sensor (in blue) and the stain gauge sensor (in red).

Since embedded sensors will be forced to flatten by the surrounding composite material, thereby eliminating sensor waviness, the sensor foils were bonded to a 1 mm thin aluminum bending beam (Loctite EA 9466, Henkel, Düsseldorf, Germany) and cured in a vacuum bag at room temperature for 24 h before the beam including sensors was subjected to another burn-in. The resistance of the SFC sensor and in parallel also the resistance of one of the strain gauge sensors aligned perpendicular to the bending axis for maximum strain sensitivity were recorded while exposed to varied temperatures in the oven. The obtained course of the strain-induced relative change in resistance ΔR/R is given in Figure 7. The strained state corresponds to a bending radius of r= 335 mm.

These measurements show that for the SFC sensors, the resistance compared to the unloaded sensor increases by 0.37% when a bending with r=335 mm is applied, whereas the strain gauge shows an increase of 0.78%, averaged over the temperature range considered. It is also worth noting that most of the variations in Figure 7 are consistent for both curves and are probably due to inaccuracies in the temperature measurement. As predicted by the simulations, the SFC temperature sensor is not completely insensitive to strain but exhibits a sensitivity to strain caused by a uniaxial bending stress, which is less than half of what is obtained with the strain gauge. The strain insensitivity of the SFC sensor in comparison to the strain gauge sensor that resulted from the experiments was somewhat weaker than predicted by the simulations. While a factor of 2.8 (resistance change of DMS vs. SFC) was observed in the simulations, a factor of 2.1 was achieved in the experiments. This is probably due to the fact that the real stress distribution in the experiments is more complex than assumed in the simulations. It is worth mentioning that a deviation in the temperature, which is made by ignoring this level of strain, is insignificant.

## 4. Conclusions

The new SFC temperature sensor can be fabricated as part of a sensor node, which contains also strain gauges and a coplanar capacity for impedance characterization in the format of an ultra-thin foil. Other metallic sensor materials such as platinum, copper, or biocompatible titanium could also be considered depending on the application. Semiconducting materials could be beneficial in terms of strain and temperature sensitivity, which unfortunately means that also the unwanted cross-sensitivities would increase. The developed sensor node can be integrated in composite material without weakening the material [18]. In simulations and also in the experiments, a strong suppression of strain influences on this type of sensor could be proven. The influence of the strain on the measurement signal is less than half of the influence obtained with linear track sensors. This type of temperature sensor can be used without strain correction if the strain and temperature profiles typically expected in curing composites can be assumed. In combination with the strain gauge half-bridge also present in the sensor node, the strain of the entire sensor can be obtained by an independent measurement, and thus, also non-negligible strains can be compensated. The complete sensor node is manufactured from few materials in a simple manner and can be evaluated by low-cost electronic components. With suitable evaluation algorithms that allow the three sensor types to mutually compensate each other for temperature influences and strain, this sensor node can represent an ideal tool for spatial and temporal monitoring of the curing process in composite components. Since the sensors are quite inexpensive to manufacture, the spatial resolution can be achieved by placing them in a large number of positions in the structure. The fact that the sensors remain in the composite also opens up interesting possibilities for structural health monitoring.

## Data Availability

The test data generated during the experiments can be requested from the authors.

## Abbreviations

The following abbreviations are used in this manuscript:

FEM	Finite element method
PTC	Positive temperature coefficient
NTC	Negative temperature coefficient
PI	Polyimide
PVDF	Polyvinylidene fluoride
PEI	Polyetherimide
CFRP	Carbon fiber-reinforced polymers
UV	Ultraviolet
SFC	Space-filling curve
